# Aerosol assisted chemical vapour deposition of gas sensitive SnO_2_ and Au-functionalised SnO_2_ nanorods via a non-catalysed vapour solid (VS) mechanism

**DOI:** 10.1038/srep28464

**Published:** 2016-06-23

**Authors:** Stella Vallejos, Soultana Selina, Fatima Ezahra Annanouch, Isabel Gràcia, Eduard Llobet, Chris Blackman

**Affiliations:** 1 SIX Research Centre, Brno University of Technology, Technická 10, Brno, CZ-61600, Czech Republic; 2 Department of Chemistry, University College London, 20 Gordon Street, London, WC1H 0AJ, UK; 3 MINOS-EMaS, Departament d’Enginyeria Electrònica, Universitat Rovira i Virgili, Paisos Catalans 26, Tarragona, 43007, Spain; 4 Aix Marseille Université, CNRS, Université de Toulon, IM2NP UMR 7334, Marseille, France; 5 Instituto de Microelectrónica de Barcelona (IMB-CNM, CSIC), Campus UAB, Barcelona, 08193, Spain

## Abstract

Tin oxide nanorods (NRs) are vapour synthesised at relatively lower temperatures than previously reported and without the need for substrate pre-treatment, *via* a vapour-solid mechanism enabled using an aerosol-assisted chemical vapour deposition method. Results demonstrate that the growth of SnO_2_ NRs is promoted by a compression of the nucleation rate parallel to the substrate and a decrease of the energy barrier for growth perpendicular to the substrate, which are controlled via the deposition conditions. This method provides both single-step formation of the SnO_2_ NRs and their integration with silicon micromachined platforms, but also allows for *in-situ* functionalization of the NRs with gold nanoparticles *via* co-deposition with a gold precursor. The functional properties are demonstrated for gas sensing, with microsensors using functionalised NRs demonstrating enhanced sensing properties towards H_2_ compared to those based on non-functionalised NRs.

Due to their size dependant properties the synthesis of nanostructures (nanowires (NWs), nanorods (NRs), nanotubes (NTs) and nanobelts (NBs)) has become one of the most active research areas within the nanoscience community. Nanostructured materials, in particular semiconducting metal oxides (SMOx), have demonstrated exceptional optical and electrical properties due to electron and phonon confinement, with higher surface-to-volume ratios, modified surface work function, higher surface reaction activity, better catalytic efficiency and stronger adsorption ability compared to their bulk counterparts^1^.

Tin oxide (SnO_2_) is an intrinsic n-type wide-bandgap SMOx with applications in transparent conducting electrodes, solar cells and gas sensors^2^,^3^,^4^. In particular SnO_2_ is used in most current commercial resistive gas sensors and is the most studied material in the gas sensing literature, with demonstrated sensitivity to carbon monoxide, hydrogen, ethanol, and nitrogen dioxide, amongst others^3^,^4^,^5^,^6^.

Nanostructures of SnO_2_ have been synthetized using various methods, including chemical vapour deposition (CVD)^7^,^8^,^9^,^10^,^11^,^12^,^13^,^14^,^15^, thermal evaporation^16^,^17^,^18^,^19^, laser ablation^20^, template (AAO – anodic alumina oxide) assisted electrodepositon^21^,^22^, and liquid-phase methods^23^,^24^. Among these, vapour-phase synthesis (e.g. CVD) offers potential advantages, including the ability to operate in continuous mode and greater material purity. However CVD routes to nanostructured SnO_2_ typically require significant amounts of energy to reach the high temperatures required to break strong chemical bonds during the precursor-to-material conversion, which can lead to incompatibilities when integrating this process with microsystems and/or temperature sensitive substrates. To avoid these stringent conditions and allow nanostructure growth at temperatures as low as 650–800 °C^15^ or 550–750 °C^8^, catalyst seeds on the substrate are used to promote a vapour-liquid-solid (VLS) growth mechanism. However, the need for extra surface pre-treatment processing steps adds potential technological limitations in device fabrication. The formation of nanostructures *via* a vapour-solid (VS) mechanism, in contrast, avoids the use of catalyst seeds and facilitates device fabrication, but the synthesis of tin oxide nanostructures *via* a VS mechanism has so far required the use of very high temperatures in the range of 850 °C and 1150 °C^10^,^11^,^13^,^14^,^25^, or the use of plasma systems to reduce the deposition temperature, both of which can damage fragile substrates^26^. Recently, however, we have recognised that aerosol assisted (AA)CVD (a variant of traditional CVD which uses aerosol droplets to transport the precursor solution to the heated reaction zone) can lead to the formation of tungsten oxide nanostructures *via* a VS mechanism at relatively low onset temperatures, attributed to formation of reactive intermediates during deposition^27^. This method, which works at atmospheric pressure and relies on a solution-based delivery approach, is advantageous over traditional CVD as it allows for a wider range of precursors to be utilised^28^. It also allows for the functionalization of SMOx nanostructures with metal nanoparticles (NPs) in a single processing step *via* co-deposition, which we have demonstrated previously for incorporation of gold^29^, platinum^30^ or other SMOx (e.g. Cu_2_O^31^ and Fe_2_O_3_^32^) nanoparticles segregated at the surface of tungsten oxide nanostructures.

There are very few reports on synthesis of tin oxide or tin oxide composites *via* AACVD, with most referring to the formation of thin films^33^,^34^,^35^,^36^,^37^,^38^,^39^,^40^ rather than nanostructures. The precursors used for the AACVD of tin oxide include common precursors used in traditional CVD^41^, such as monobutyltin trichloride (C_4_H_9_SnCl_3_)^38^,^40^, and tin(II) chloride dihydrate (SnCl_2_·2H_2_O)^33^,^42^, tin complexes [Sn(18-Cr-6)Cl_4_]^34^ and [Sn(H_2_O)_2_Cl_4_](18-Cr-6)^34^, or for the deposition of composite films bimetallic complexes such as C_29_H_40_FeO_2_Sn^37^, C_35_H_28_FeO_2_Sn^37^, or [ZnSn(dmae)(dmaeH)_2_(NO_3_)Cl_4_]·2H_2_O^39^.

Here we demonstrate the AACVD of SnO_2_ nanostructures, in the form of NRs, from a simple commercial tin precursor (SnCl_4_·5H_2_O) at atmospheric pressure and exceptionally reduced process temperatures compared to existing VS-based CVD methods. In addition, to enhance the functionality of tin oxide we use the co-deposition opportunities afforded by AACVD to incorporate, in the same single-step, Au NPs at the surface of tin oxide NRs. These non-functionalised (SnO_2_) and functionalised (Au@SnO_2_) nanostructures are directly integrated into silicon micromachined platforms (fabricated using Micro-Electro-Mechanical-System (MEMS) technology) and validated for gas sensing, with sensors based on Au-functionalised SnO_2_ NRs demonstrating enhanced properties.

## Results

### AACVD of SnO_2_

**Figure 1:**
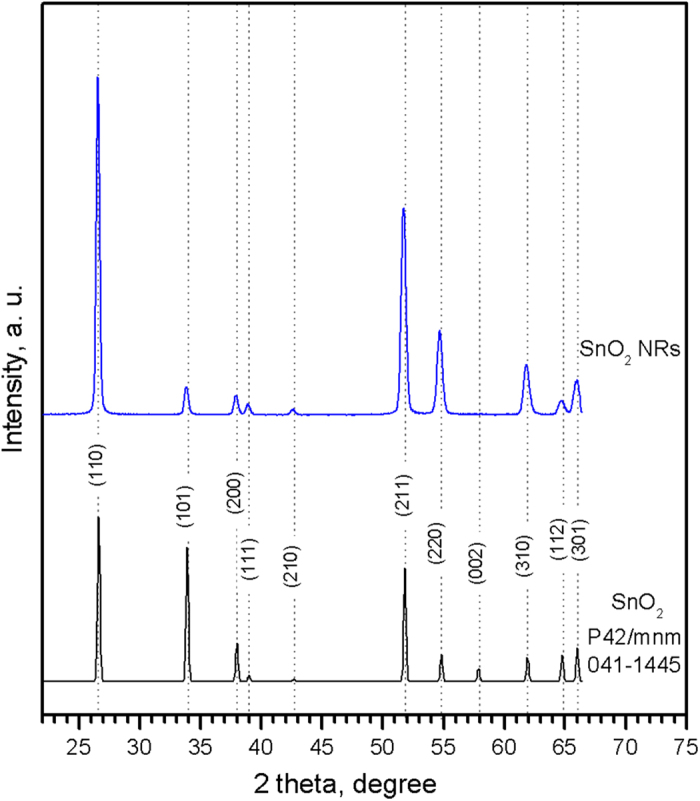
Typical XRD pattern of the SnO_2_ NRs deposited at temperatures between 300 and 620 °C from a solution of SnCl_4_·5H_2_O dissolved in acetone. The diffraction peaks in the data can be indexed to a tetragonal phase (P42/mnm, ICCD card no. 041-1445) of tin oxide.

AACVD of SnCl_4_·5H_2_O dissolved in acetone or methanol at temperatures between 300 and 675 °C resulted in the formation of adherent uniform greyish films on silicon wafers. XRD analysis confirmed the presence of tetragonal SnO_2_ (P42/mnm, *a* = 4.7380 Å, *c* = 3.1870 Å, literature P42/mnm space group, *a* = 4.7382 Å, *c* = 3.1871 Å; ICCD card no. 41-1445) for all the films (Fig. 1), except for those deposited from a methanol-based solution at temperatures exceeding 500 °C (Fig. S1), which showed extra diffraction peaks corresponding to metallic tin (I41/amd, *a* = 5.8310 Å, *c* = 3.1820 Å, ICCD card no. 4-673).

**Figure 2:**
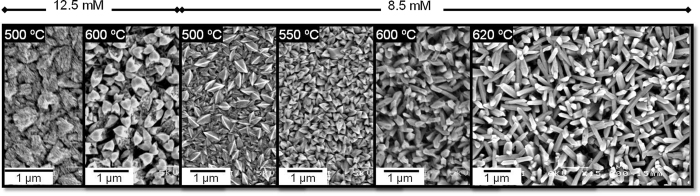
Film morphology observed by SEM images at different solution concentrations and various deposition temperatures.

The morphology of the films was strongly dependent on the deposition temperature, solution concentration and solvent. For instance (Fig. 2), the films deposited from acetone-based solution (12.5 mM) displayed uneven morphology at 500 °C and porous structures with twinned crystallites at 600 °C, whereas the films deposited from lower solution concentration (8.5 mM) showed the formation of porous films with defined crystallites at 500 °C, a high density of leaf-like structures at 550 °C, and the formation of a high density of NRs at 600 °C. An increase in temperature from 600 °C to 620 °C improved the definition of the NRs, and above this temperature (up to 675 °C) the structures suffered deformations with less material deposited on the substrate, most likely due to homogeneous gas-phase reaction resulting in the formation of powder and the action of thermophoretic forces. A further decrease of solution concentration to 4.3 mM ended-up without visible film deposition. Similar test were carried out for methanol-based solutions, which produced porous films for solution concentrations of 12.5 and 8.5 mM in the temperature range of 300 °C and 675 °C (Fig. S2), and no visible deposition when the concentration was decreased to 4.3 mM.

Estimation of the energy activation for the perpendicular growth (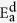) of the films^43^ *via* the Arrhenius equation indicated lower 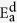 for films grown from acetone-based solutions (16.2 kJ/mol) compared to films grown from the methanol-based solutions (27.4 kJ/mol), the latter being in agreement with the value reported in the literature (24.12 kJ/mol)^44^ for the CVD of planar SnO_2_ films from SnCl_4_·5H_2_O.

### SnO_2_ NRs

**Figure 3:**
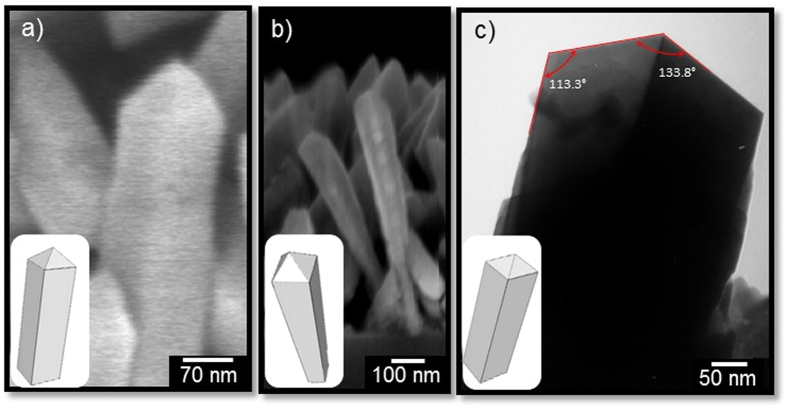
Cross-section SEM (**a**,**b**) and TEM (**c**) images of SnO_2_ NRs grown at 620 °C from SnCl_4_·5H_2_O dissolved in acetone (8.5 mM). The insets show the NR view models (not to scale).

Detailed examination of the NRs formed at 620 °C *via* AACVD suggest that the structures have a prism-like morphology terminated in a pyramidal cap (Fig. 3a), with a total length of ~700 nm and apparently wider sides at the highest part of the prism (~100 nm) in contrast to the base (Fig. 3b), corresponding to an aspect ratio of approximately 7. TEM imaging of the NRs end-cap indicates a similar morphology to that observed by SEM with an apex and lateral angle of 133.8° and 113.3°, respectively (Fig. 3c). These geometrical features are consistent with the models reported previously on the study of octahedral SnO_2_ structures synthesized from SnCl_4_·5H_2_O *via* a hydrothermal process^45^, and suggest that the pyramidal caps and the prism-like base might be enclosed by the {111} and {110} facets, respectively.

**Figure 4:**
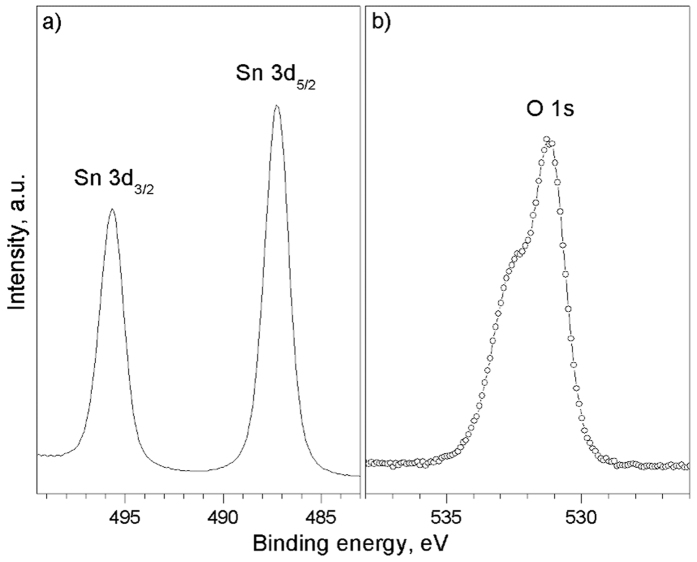
XPS spectrum of the SnO_2_ NRs grown at 620 °C from SnCl_4_·5H_2_O dissolved in acetone (8.5 mM).

The Sn 3d region of the XPS spectra for the SnO_2_ NRs (Fig. 4a) exhibited Sn 3d_5/2_ and Sn 3d_3/2_ doublets for binding energies of ~486.3 and ~494.7 eV, respectively, confirming the presence of the Sn^4+^ oxidation state^46^,^47^. In addition, the good symmetry of the peaks, showing no sub-components, suggests the absence of Sn^2+^. The O 1s core level (Fig. 4b), in contrast, indicates the presence of two components, with the main peak at ~531 eV, assigned to the lattice oxygen, and a shoulder at ~532.5 eV, which is assigned to contamination (organic fragments) on the samples in light of the lack of additional structures on the Sn 3d core level^46^. Both WDX and XPS analysis showed no evidence of chlorine contamination and similar tin contents (anal. calcd. for SnO_2_: Sn 33.27 and O 66.53 at.%, found WDX: Sn 30.18 at.%, found XPS: Sn 30.30 at.% with a O/Sn ratio of 1.9 taking into account only the component assigned to lattice oxygen). The formation of SnO_2_ at this temperature is consistent with the TGA of SnCl_4_·5H_2_O, which has been shown to produce nearly stoichiometric tin oxide from 450 °C to 700 °C^41^,^48^.

### Au@SnO_2_ NRs

**Figure 5:**
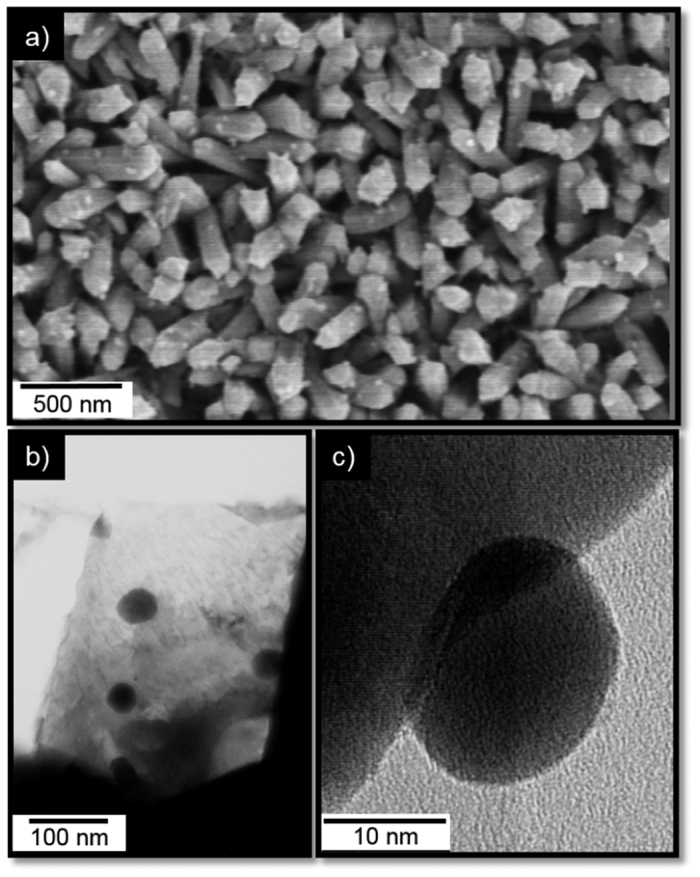
SEM (**a**) and TEM images at low (**b**) and high (**c**) magnification for the Au@SnO_2_ NRs.

The films synthesised at 620 °C by co-deposition *via* AACVD of tin and gold precursors dissolved in acetone resulted in the formation of adherent uniform greyish films on silicon substrates, with similar appearance to those deposited from only the tin precursor. SEM imaging of the Au@SnO_2_ films revealed a high density of NRs (Fig. 5a) with similar features to those observed for intrinsic SnO_2_ films deposited at 620 °C (Fig. 2), though with apparently small particles dispersed at the surface. Similarly, TEM imaging of the functionalised NRs displayed NPs along the NR surface (Fig. 5b c), which indicate the incorporation of Au NPs as noticed previously when co-depositing gold with tungsten oxide nanoneedles^29^. The NPs showed spherical morphologies and sizes up to 35 nm; an analysis of the size distribution of these particles *via* TEM was complex due to NRs tending to agglomerate on the TEM grids and the relatively high thickness of NRs.

**Figure 6:**
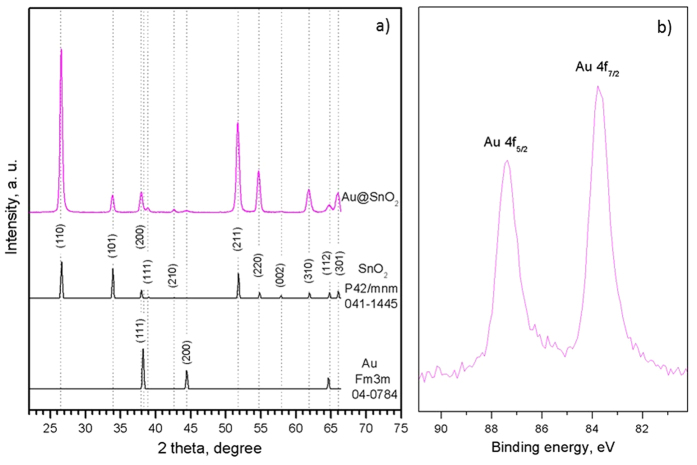
Typical XRD pattern of the Au@SnO_2_ NRs (**a**) indexed to a tetragonal phase (P42/mnm, ICCD card no. 041-1445) of tin oxide and to the gold face centred cubic phase (Fm3m, ICCD card no. 04-0784). Au 4f core-level spectrum recorded on Au-functionalised tin oxide NRs (**b**).

XRD of the Au@SnO_2_ NRs (Fig. 6a) revealed similar pattern than that observed for intrinsic SnO_2_ NRs (Fig. 1) showing a tetragonal phase (P42mnm space group, *a* = 4.7382 Å, *c* = 3.1871 Å; ICCD card no. 411-1445), with an extra weak diffraction at 44.3 degrees corresponding to the (200) reflections of gold face centred cubic phase (Fm3m space group, *a* = 4.07860 Å; ICCD card no. 04-0784). XPS of the films indicated a (0.9 at.%) 3.7 wt.% Au in the films with the characteristics of Au 4f core level spectra being in agreement with that reported for gold metal^29^, which suggests the gold NPs incorporated at the surface of the tin oxide NRs are in the metallic state. The Au:Sn ratio determined by XPS (found: Au:Sn 3.22 at.% (5.23 wt.%)) and compared to the initial ratio present in the precursor solution used for AACVD (anal. calcd for Au:Sn 23.2 wt.%) showed the incorporation of gold NPs is about 23% efficient, which is higher compared to the efficiency (5%) obtained previously for the co-deposition of tungsten oxide and gold^30^, likely due to the higher temperature of deposition.

### SnO_2_ and Au@SnO_2_ NRs integrated into micromachined platforms

**Figure 7 f7:**
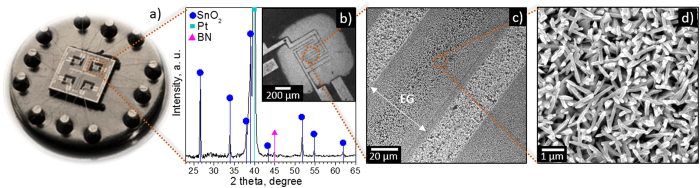
Photograph of the 4-microsensor array mounted on a standard TO-8 package (**a**), XRD of a single microsensor based on SnO_2_ nanorods (platinum and boron nitride diffractions come from the microsensor platform) (**b**) and SEM images at low (**c**) and high (**d**) magnification of the SnO_2_ material grown onto the microplatform (EG represents the electrode gap).

The SnO_2_ and Au@SnO_2_ films grown directly into the micromachined platforms (Fig. 7a) *via* AACVD at 620 °C, showed similar diffraction patterns (Fig. 7b) with NR-like morphology (Fig. 7c[Fig f7]d) compared to that observed for the same deposition conditions on silicon wafers (**section SnO_2_ NRs** and **Au@SnO_2_ NRs**). Measurements of the electrical resistance of the films using the microelectrodes confirmed a good electrical contact, with the resistance at different sensor operating temperatures showing a direct dependency of the conductivity to the temperature, as expected for an n-type semiconductor. The apparent energy activation for electrical conduction (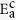) estimated for temperatures between 250 °C and 350 °C yielded a value of 0.35 eV for the SnO_2_ NRs, which is consistent with the 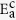 for porous SnO_2_ (between 0.28 eV and 1.1 eV) reported previously^49^. The 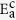 estimated for the Au@SnO_2_ NRs, in contrast, yielded a much lower value (0.11 eV), approximately 69% less than the obtained with the SnO_2_ NRs. This change is much higher compared to that recorded previously for the co-deposited Au-functionalised tungsten oxide (Au@WO_3_) structures, which showed only 9% lower 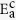 compared to the non-functionalised tungsten oxide structures^30^. These numbers show a close relation with the theoretical differences in the work functions (W_F_) of these materials, as the W_F_ of Au (4.8 eV)^50^ is 16% and 2% lower than that of SnO_2_ (5.7 eV)^2^ and WO_3_ (4.9 eV)^51^, respectively, and the relative value of these differences (i.e. 8) is similar to that found for the 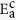 of Au@SnO_2_ and Au@WO_3_ (i.e. 7.7).

### Gas sensing characterization

**Figure 8:**
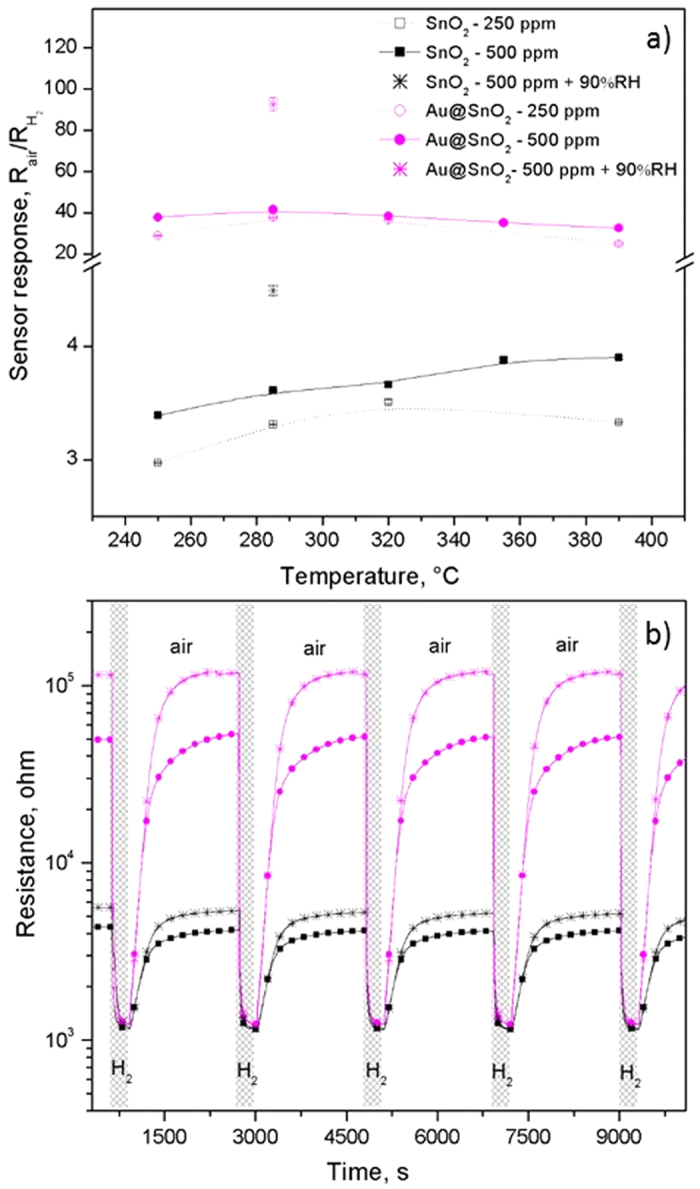
Sensor responses to 250 and 500 ppm of H_2_ as a function of the operating temperature (**a**) and film-resistance changes towards 500 ppm of H_2_ at 290 °C (**b**). The response to 500 ppm of H_2_ in humidity (90% RH) is displayed in both plots.

Gas sensing tests were carried out to H_2_ and CO at various operating temperatures between 250 and 390 °C using dc resistance measurements. The temperature dependency of the sensor response for each analyte and concentration is displayed in Fig. 8a and S3a. Results for H_2_ suggest a similar trend for sensors composed of SnO_2_ and Au@SnO_2_ NRs, with slight changes of the response by increasing the operating temperature, whereas results for CO display a tendency to increase the sensor response by increasing the operating temperature, particularly for sensors comprised of Au@SnO_2_ NRs. Figure 8a and S3a also reveal greater sensor responses for the Au-functionalised NRs compared to the intrinsic NRs, with higher increments (12-fold) for H_2_ than for CO (2-fold), which reduces the cross-response of these analytes and in turn improves the selectivity of the device. The typical change of resistance recorded for each type of sensor is shown in Fig. 8b and S3b. Overall, the sensor responses displayed an n-type behaviour, i.e. decreasing electrical resistance when exposed to H_2_ or CO. During the testing period (i.e. 100 h) the sensor response showed good reproducibility with standard errors below ±1.5%, and little variation of the baseline resistance at each operating temperature (below ±6%), with the SnO_2_ NRs showing lower baseline resistances compared to the Au@SnO_2_ NRs (e.g. 4.5 kΩ for SnO_2_ and 50 kΩ for Au/SnO_2_ at 290 °C). With the injection of humidity (90% RH) in the system, at operating temperature of 290 °C, the baseline resistance of the sensors increased up to 5.5 kΩ for sensors based on SnO_2_ NRs and 120 kΩ for those based on Au@SnO_2_ NRs. Under these conditions the electrical resistance changes to H_2_ remained stable and reproducible, but the sensor response was higher compared to the response obtained in dry air (Fig. 8), showing an increase of the sensor response to H_2_ up to 18% for the SnO_2_ NRs and 140% for the Au@SnO_2_ NRs, indicating decreased humidity tolerance of the SnO_2_ NRs after decoration with gold NPs.

**Figure 9:**
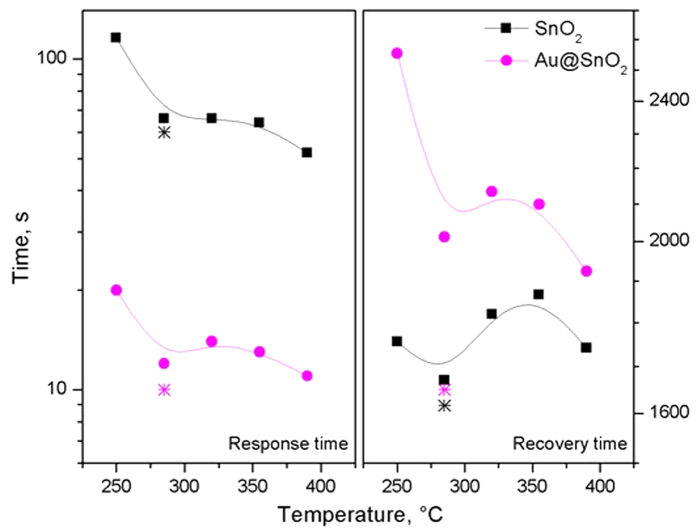
Response (**a**) and recovery (**b**) time for each type of sensor towards 500 ppm of H_2_. The response and recovery times at 290 °C in humidity are also shown in the plot and represented by a star.

A detailed view of the normalised response to H_2_ at 290 °C for the sensors based on SnO_2_ and Au@SnO_2_ NRs show the characteristics of the response (t_R_) and recovery (t_rec_) time, and the time needed to reach stationary state when exposed to H_2_ (Fig. S4). The overall view of the response and recovery times as function of the temperature for each type of sensor towards H_2_ (Fig. 9) suggests a decrease in the response and recovery time of the sensor as the operating temperature increases. Results in Fig. 9 also show faster response times for the Au@SnO_2_ NRs compared to intrinsic NRs, and an inverse relationship of the recovery time with respect to the response time, i.e. longer recovery times for Au@SnO_2_ as opposed to SnO_2_. Similar comparison between the non-functionalised and Au-functionalised tungsten oxide structures studied previously showed a similar tendency of the response/recovery time^30^.

After 100 h of testing the sensor alternately to H_2_ and CO in dry and humid environment and at different operating temperatures, the gas sensitive nanostructures where examined again using SEM and XRD. In comparison to the initial samples the morphology of the SnO_2_ and Au@SnO_2_ NRs was unchanged and the diffraction patterns were identical, indicating a good stability of the devices.

## Discussion

The AACVD of SnCl_4_·5H_2_O allowed for the synthesis of nanostructured SnO_2_ films by adjusting the deposition temperature, the precursor concentration and solvent used to produce the aerosol. The ideal conditions for growing SnO_2_ NRs were found using a solution 8.5 mM of SnCl_4_·5H_2_O dissolved in acetone at 620 °C (Fig. 2), a much lower temperature compared to other CVD methods based on VS mechanism which require temperatures exceeding 850 °C^10^,^11^,^13^,^14^,^25^. The AACVD of SnO_2_ NRs *via* VS mechanism at this relatively low temperature can be connected to two factors: a drop of the energy activation for the perpendicular growth of the film registered when using acetone (16.2 kJ/mol) instead of methanol as carrier solvent, and a decrease of the number of precursor molecules per volume induced by reducing the initial solution concentration about 30%. This is consistent with previous analysis of the growth mechanism of tungsten oxide nanorods *via* AACVD, which indicated that the transition from planar to nanorod films (characterised by the Frank-van der Merwe and Volmer-Weber growth mode, respectively) at a fixed temperature requires the attenuation of these factors (i.e. 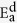 and density of precursor molecules)^43^. As the same deposition temperatures and AACVD parameters (e.g. flow, gas carrier, solution concentration) for the different systems were kept constant with the exception of the ‘carrier’ solvent, it is therefore reasonable to suggest that the use of solvents such as acetone and methanol, which can decompose *via* radical mechanism at these temperatures^52^,^53^,^54^, might also add active reactive products to the reaction in AACVD. This has a direct influence on the decomposition profile of the tin precursor and in turn modifies the energy activation for the perpendicular growth rate of the film promoting formation of nanostructured growth. The presence of metallic tin at 600 °C when using a methanol solution (Fig. S1) corroborates this hypothesis, as this suggests a different chemical species, with a different decomposition profile to the initial precursor, is involved in the AACVD process when this solvent was used. Acetone may also play a similar role in the decomposition mechanism of the tin precursor but results suggest it is less reactive than methanol. The change in reactive chemical species is likely to affect the reaction time and hence the ratio between this and the reactor residence time, parameters which have previously been shown to be involved in the transition from planar to columnar-like structures^55^.

The incorporation of a second precursor (HAuCl_4_·3H_2_O) during the AACVD of SnO_2_ NRs allowed for the co-deposition of Au@SnO_2_ NRs, similar to our previous results for the co-deposition of gold NPs with tungsten oxide nanostructures^29^,^30^, although requiring use of a different carrier solvent and higher deposition temperatures (acetone at 620 °C instead of methanol at 400 °C for tungsten oxide). Overall, the analysis of these structures showed that the gold NPs incorporated at the tin oxide NRs are in metallic state (Fig. 6b) with no evidence of modification of either the morphology (Fig. 5) or the crystalline structure (Fig. 6a) of the NRs. However, the lower energy activation of conduction (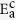) estimated for the Au@SnO_2_ samples (0.11 eV) compared to that of the intrinsic SnO_2_ (0.35 eV) may suggest the presence of Au-O bonds at the surface of the NPs, as inferred earlier for the co-deposited gold NPs on tungsten oxide^30^.

The direct integration of these structures (SnO_2_ and Au@SnO_2_) with micromachined platforms was achieved, demonstrating this process and the associated temperature are compatible with the complementary electronics on these devices. The capability to integrate nanostructured materials with microsystems, such as those used in this work (i.e. fabricated by MEMS technology), represents a technological advantage for gas sensing, as MEMS sensor platforms provide low power consumption features and are suitable for integrating monolithic sensor arrays. Sensor testing showed an optimum operating temperature for SnO_2_ of 300 °C, a temperature frequently reported for SnO_2_ when sensing H_2_ and CO, Table 1. At this temperature (i.e. 300 °C) the micromachined sensors registered 16-times less power consumption (32 mW) than the traditional planar ceramic sensors (525 mW) used in our previous works for tungsten oxide^29^.

Overall, validation of these devices towards the detection of H_2_ and CO showed good performance with stable signal, very low drift of electrical resistance over the testing period and relatively fast response. These characteristics are highly enhanced compared to our previous sensors based on SnO_2_ NPs synthesised via AACVD of tin complexes^34^; which showed a very slow and low resistance change to NO_2_ and non-sensitivity towards H_2_ or CO. A direct comparison of our results and those of the literature is relatively complex, as the performance of the sensor is not only linked to the material properties, but also in part to the characteristics of the transducers and test conditions (e.g., operating temperatures, flows, and gas concentrations). Despite this we believe that Table 1 can still be useful to evaluate the tendency of SnO_2_ towards the analytes tested in this work. Consequently, this comparative table suggests that the SnO_2_ NRs synthesised *via* AACVD provide higher sensitivity (S) to H_2_ compared to that recorded in other works for this analyte. In contrast, the sensitivity obtained for CO reveals a much lower value with respect to that observed for hydrothermal synthesised grains, although marginally higher than that reported for electrospun tin oxide fibres. These differences could be linked to some technological characteristics of each sensor, for instance the use of platinum top electrodes in the sensor architecture of the electrospun films, or the use of transfer steps and most likely the introduction of impurities when integrating the tin oxide films synthesized *via* hydrothermal or thermal evaporation method into the sensor platforms. In fact, impurities such as chlorine (typically introduced from the precursors), or potassium and calcium (often introduced by the use of transfer steps) have been demonstrated to play a relevant role in the surface activity and sensing properties of tin oxide^56^; most of the tin oxide films in Table 1 were synthesised from chlorine containing precursors (i.e. SnCl_4_ and SnCl_2_) or integrated using transfer steps. The analysis of our SnO_2_ NRs integrated directly on the micromachined platform though showed no evidence of chlorine, likely due to easy elimination *via* HCl from the SnCl_4_·5H_2_O precursor.

The shape and geometry of the AACVD NRs showed similar characteristics to those reported previously in prism-like rods^45^, suggesting a surface likely dominated by the SnO_2_ {110} facets. The presence of these facets, which have shown to be less favourable for CO adsorption due to the need of a particular adsorption geometry with the C-end orientated to the surface^2^, may be responsible for the attenuation of the response to CO compared to H_2_. No equivalent studies were found for H_2_, however as the adsorption of H_2_ includes the formation of intermediate molecules as water, and these have shown more favourable adsorption on SnO_2_{110} surfaces^2^, the higher responses registered for H_2_ in dry and humid air seem consistent.

The functionalization of the SnO_2_ NRs with Au NPs showed enhanced sensing characteristics, compared to the intrinsic SnO_2_ NRs, which include higher sensor response, (almost 12- and 2-times more for H_2_ and CO respectively) and a reduction of the response time of about 6 times. However, the functionalization also showed a tendency to reduce the sensor tolerance to humidity, inducing a larger change of the response to hydrogen in humid ambient compared to the non-functionalised SnO_2_ NRs. This characteristic is undesirable in gas sensing and seems to be related to both the propensity of SnO_2_ to adsorb hydroxyl groups on the surface at the temperature used for this test^57^, and the amplification of the response by analogous mechanisms than those involved in the enhancement of H_2_ and CO sensing. The mechanisms that lead the metal NPs functionalised SMOx to an enhanced gas sensing performance has been discussed in the literature previously, and generally involve surface dependent effects (i.e. chemical sensitization, which may include mechanisms such as spill-over and complementary decomposition) and/or interface dependent effects (electronic sensitization, which may include the modulation of potential barrier heights, carrier injection and conduction channel modulation)^58^,^59^,^60^. The faster response in the Au@SnO_2_ compared to the SnO_2_ NRs gives evidence of a chemical sensitization, with the Au NPs most likely accelerating the dissociation of hydrogen molecules into H atoms and simultaneously inducing a faster saturation of the active sites on the NR surface *via* spill-over. This saturation (also observed in the response (Fig. S4) of the sensors based on Au@SnO_2_ NRs as opposed to those based on SnO_2_ NRs) is responsible for the larger desorption time required to recover the baseline resistance at each temperature (Fig. 9). In contrast, the lower 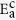 obtained for the Au@SnO_2_ NRs compared to the SnO_2_ NRs indicates an electronic sensitization, with the Au NPs facilitating the carrier injection and thus modulating the conduction channel along the nanostructure.

The ratio of the response to H_2_ and CO for each microsensor (ΔR = 2.5 for SnO_2_ and ΔR = 20 for Au/SnO_2_) indicates relatively higher cross-sensitivities for the microsensors comprised of intrinsic SnO_2_ NRs, as opposed to those comprised of Au@SnO_2_ NRs, which potentially improves selectivity to H_2_. A comparison of these ratios with those recorded for similar systems synthesised *via* sol-gel in the literature^61^ revealed similar values for the SnO_2_ NRs and nearly 7 times higher values for the Au@SnO_2_ NRs, suggesting the functionalisation of SnO_2_ with Au NPs *via* AACVD is effective for improving the selectivity of tin oxide towards H_2_ and CO.

In summary, these results demonstrate the AACVD of SnO_2_ NRs *via* VS mechanism at exceptionally reduced process temperature compared to existing CVD methods with no need for substrate pre-treatment. This allowed for the direct integration of these nanostructures into micromachined platforms and their use for sensing H_2_ and CO. The incorporation of Au NPs at the surface of the SnO_2_ NRs *via* co-deposition improved the functionality of SnO_2_, particularly to H_2_, reducing the cross-sensitivity of this analyte to CO.

## Conclusions

SnO_2_ NRs with an aspect ratio of approximately 7 were synthesized without catalyst seeds *via* AACVD of SnCl_4_·5H_2_O at 620 °C, a much lower onset temperature compared to other CVD methods based on a VS mechanism, which typically requires temperatures exceeding 850 °C. The evolution of nanorod SnO_2_ is linked to an increase in energy activation of the perpendicular growth of about 40% respect to that observed for non-nanostructured SnO_2_ films, and is attributed to the use of acetone as solvent carrier, and a reduction of precursor concentration. Co-deposition of Au NPs (<35 nm) and SnO_2_ NRs *via* AACVD was also achieved at 620 °C. The gas microsensors based on the intrinsic SnO_2_ and Au@SnO_2_ NRs were validated towards H_2_ and CO and show sensing properties that are in agreement with the literature, with notable enhancement of sensing properties for Au@SnO_2_ NRs which showed 12-fold higher response with 6-fold faster response and improved selectivity to H_2_ compared to the gas sensors based on intrinsic SnO_2_ NRs.

## Experimental Section

### SnO_2_ synthesis

SnO_2_ nanostructures were deposited at temperatures between 300 and 675 °C *via* aerosol assisted (AA) CVD of tin (IV) chloride pentahydrate (SnCl_4_·5H_2_O, Sigma-Aldrich, ≥98%) dissolved in 15 ml of acetone. In order to vary the concentration of the solution, three different weights (45, 30 and 15 mg) of SnCl_4_·5H_2_O were used for the deposition. A piezoelectric ultrasonic atomiser (Liquifog, Johnson Matthey) operating at 1.6 MHz was used to generate an aerosol of the solution. The aerosol droplets were transported to the heated substrate by a nitrogen (BOC, oxygen free) gas flow (200 cm^3^·min^−1^), and the time taken to transport the entire volume of solution was typically 15 minutes.

### Au@SnO_2_ synthesis

Nanocomposites composed of gold NPs and tin oxide NRs were synthesised at 620 °C by co-deposition, *via* AACVD, of tin (IV) chloride pentahydrate (30 mg, SnCl_4_.5H_2_O, Sigma-Aldrich, ≥98%) and tetrachloroauric acid trihydrate (4.2 mg, HAuCl_4_·3H_2_O, Sigma-Aldrich, 99.9%) dissolved in acetone (15 ml, Sigma-Aldrich, ≥99.6%) using the same system described above and following the method reported in literature^29^.

### Substrate and micromachined platforms

Silicon wafers (10 mm × 10 mm × 0.37 mm) and KBr disks were used as substrates for film analysis, whereas micromachined platforms consisting of an array of four SiO_2_/Si_3_N_4_/SiO_2_ membrane, each of them with isolated polysilicon heaters and platinum electrodes (gap: 50 μm, thick: 0.2 μm), were used for gas sensor fabrication. After deposition of the sensing active film the platforms were mounted on a TO8 package. The sensor technology was described in detail previously^62^.

### Film analysis

The morphology of the samples was examined using Scanning Electron Microscopy (SEM — Jeol 6301F, 5 keV). The structure using X-Ray Diffraction (either XRD — Bruker, AXD D8- Discover for the films grown on silicon wafers or Rigaku Smartlab 9 kW for the films grown on micromachined platfoms) and the chemical composition using Wavelength Dispersive X-Ray (WDX — Philips, XL30ESEM) and X-ray Photoelectron spectroscopy (XPS) (Thermo Scientific K-Alpha, using Al Ka radiation operated at 0.6 eV with electron gun operating at 1 eV and argon-ion gun operated at 10 eV; the binding energies were calibrated to the C 1s peak at 284.5 eV). TEM (JEOL JEM-100CX II, 100 kV) images were carried out on samples prepared by deposition on KBr substrates followed by dissolution of the substrate in distilled water and suspended on Cu grids.

### Gas sensing tests

Gas sensors were tested in a continuous flow (200 sccm) test chamber (280 cm^3^) as previously described^62^. The sensors were exposed to 250 and 500 ppm of hydrogen and carbon monoxide for 10 min and subsequently the chamber purged with air until initial baseline resistance was recovered. The whole testing period comprised of 100 h during which sensors were tested to different hydrogen and carbon monoxide concentrations at operating temperatures between 250 and 390 °C in dry and humid ambient, performing 5 replicates for each condition. To obtain the desired analyte concentration calibrated cylinders of either hydrogen (Praxair, 1000 ppm) or carbon monoxide (Praxair, 1000 ppm) were mixed with pure synthetic air (Carburos Metálicos, 99.99%) by means of a mass flow system (Bronkhorst hi-tech 7.03.241). The sensor response was defined as R = R_a_/R_g_, where R_a_ is the sensor resistance in air at stationary state and R_g_ represents the sensor resistance after 10 min of the analyte exposure. The response time was defined as the time required for the sensor to reach 90% of the sensor response, and the recovery time as the time required to reach 10% of the initial baseline resistance after the analyte was purged.

## Additional Information

**How to cite this article**: Vallejos, S. *et al*. Aerosol assisted chemical vapour deposition of gas sensitive SnO_2_ and Au-functionalised SnO_2_ nanorods via a non-catalysed vapour solid (VS) mechanism. *Sci. Rep.* **6**, 28464; doi: 10.1038/srep28464 (2016).

## Supplementary Material

Supplementary Information

## Figures and Tables

**Table 1:** Summary of the gas sensing properties of non-functionalised tin oxide reported in the literature for H_2_ and CO.

SnO_2_	Method	Integration	Sensor platform	T_op_ °C	Gas	C ppm	R	t_res_ s	t_rec_ s	S %	Ref
**NRs**	**AACVD**	**direct**	**μM**^**P**^	**290**	**H**_**2**_	**250**	**3**.**3**	**70**	**1700**	**0**.**20**	**This work**
Grains	HT	transfer	Ceramic^P^	400	H_2_	2000	26	10	180	0.16	61
Grains	CVD	direct	Ceramic^P^	300	H_2_	100	1.03	–	–	–	63
NRs	TE	transfer	Si-based^P^	200	H_2_	250	1.9	–	–	0.17	64
**NRs**	**AACVD**	**direct**	**μM**^**P**^	**290**	**CO**	**250**	**1**.**1**	–	–	**0**.**04**	**This work**
Grains	HT	transfer	Ceramic^T^	300	CO	200	3	7.2	10.2	0.17	65
NWs	CVD	direct ^seeds^	Ceramic^P^	–	CO	400	3.9	10	–	–	66
fibers	ES	direct	Si-based^P^	300	CO	10	6	–	–	0	67

NRs: nanorods, NWs: nanowires, HT: hydrothermal, TE: thermal evaporation, ES: electrospinning, seeds: gold catalytic seeds for the NW growth, μM: micromachined, P and T: planar and tubular architecture, T_op_: sensor operating temperature, C: concetration, R: response (R_air_/R_gas_), t_res_: response time, t_rec_: recovery time, S: sensitivity estimated from each referenced work and defined as the ratio between the change in sensor response for a fixed change in analyte concentration.
