# Supplementary material for: Aerosol assisted chemical vapour deposition of gas sensitive SnO2 and Au-functionalised SnO2 nanorods via a non-catalysed vapour solid (VS) mechanism
Source: Sci Rep. 2016 Jun 23;6:28464. doi: 10.1038/srep28464 (PMC4917840; doi:10.1038/srep28464)
Supplement: Supplementary Information [file srep28464-s1.pdf]

SUPORTING INFORMATION

Aerosol assisted chemical vapour deposition of gas sensitive SnO_2 and Au-functionalised SnO_2 nanorods via a non-catalysed vapour solid (VS) mechanism

Stella Vallejos,[1]* *Soultana Selina,*[2] *Fatima Ezahra Annanouch,*[3] *Isabel Gracia,*[4] *Eduard Llobet,*[3] *Chris Blackman*[2]*

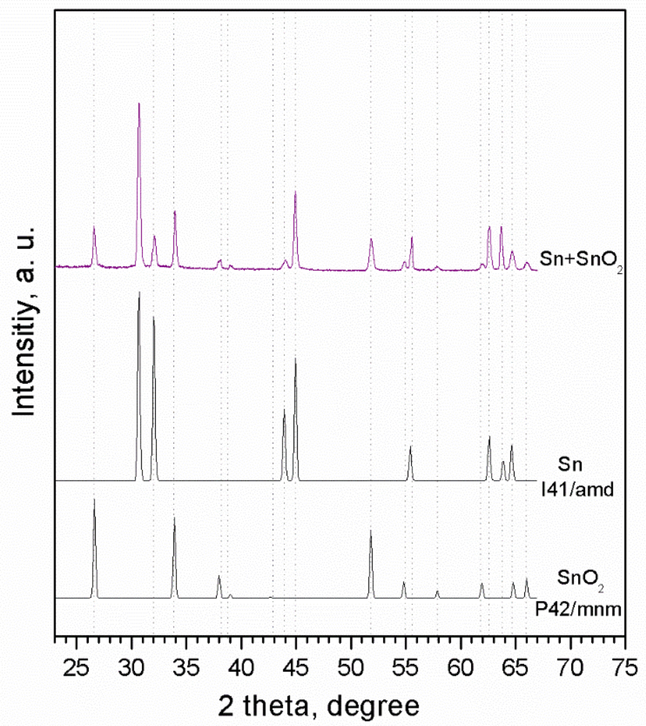

Figure S1. Typical XRD pattern of the aerosol assisted chemical vapor deposited films at 600 °C from methanol-based solution. The diffraction peaks in the data can be indexed to a tetragonal phase (P42/mnm) of tin oxide (ICCD card no. 041-1445) and a body-centered tetragonal phase (I41/amd) of metallic tin (ICCD card no. 004-0673)

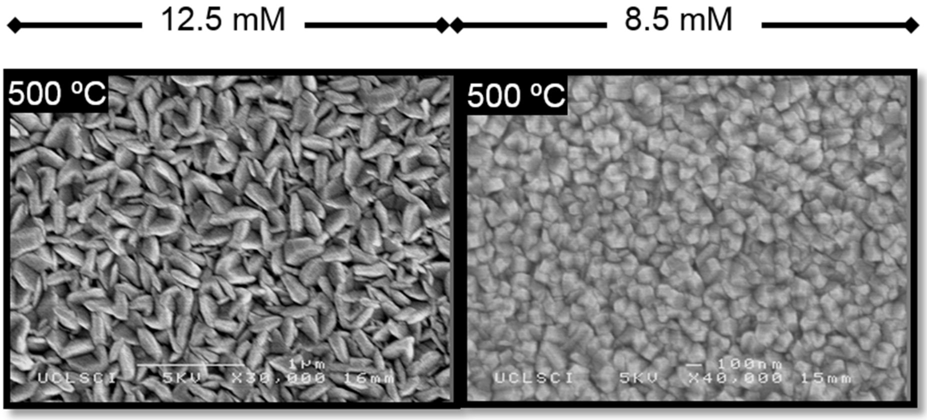

Figure S2. SEM imaging of the films deposited at 500 C from a methanol-based solution with different precursor concetration.

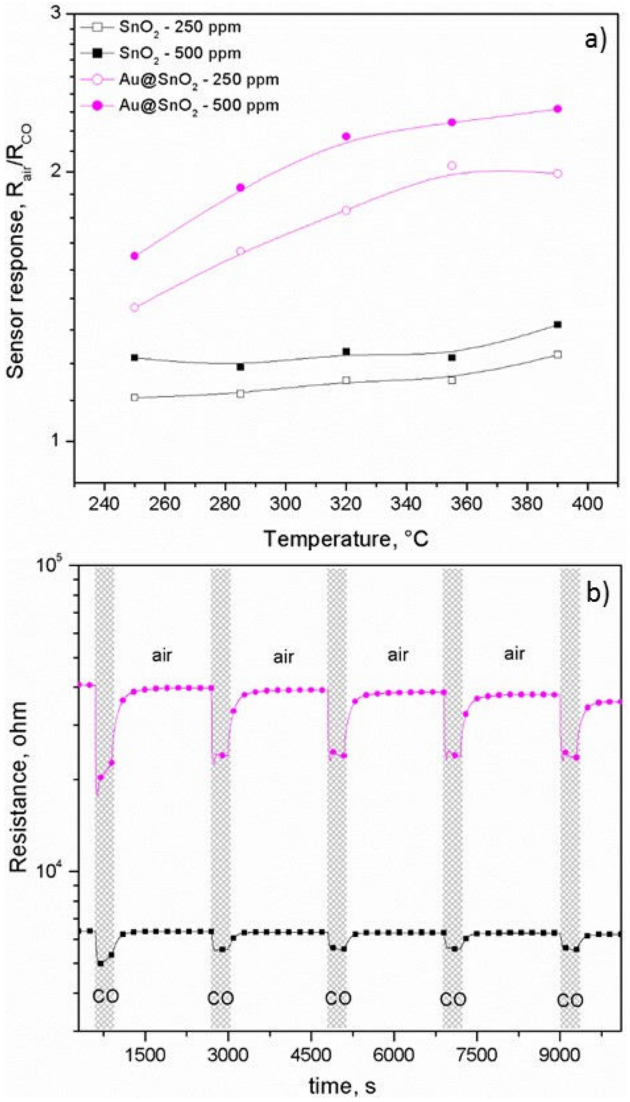

Figure S3. Sensor responses to 250 and 500 ppm of CO as a function of the operating temperature (a) and film-resistance changes towards 500 ppm of CO at 290 °C (b).

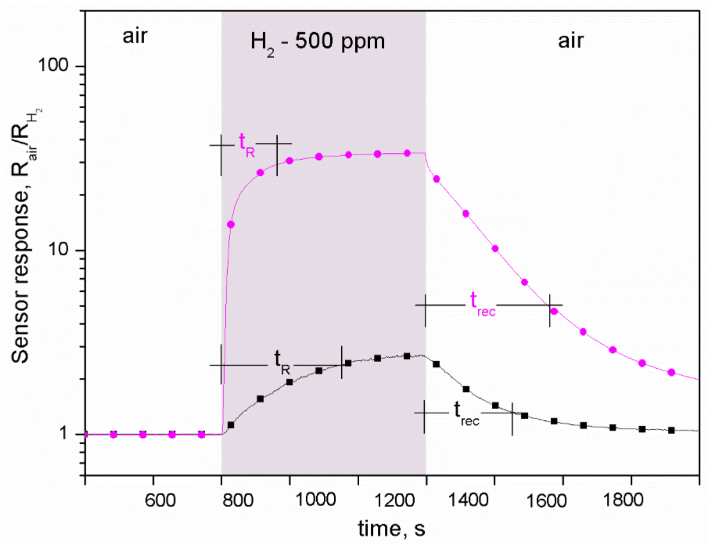

Figure S4. Normalised sensor response for 500 ppm of H_2 (a) and CO (b) measured at 290 °C. t_R and t_{rec} represent the response and recovery time, respectively.
